# Identification of Fungi Causing Root Rot in Oregano Crops in Southern Peru: Morphological and Molecular Analysis

**DOI:** 10.3390/pathogens14080746

**Published:** 2025-07-29

**Authors:** Rubí Adelin Quispe-Mamani, Liduvina Sulca-Quispe, Wilson Huanca-Mamani, Mirna G. Garcia-Castillo, Patricio Muñoz-Torres, German Sepúlveda-Chavera

**Affiliations:** 1 Laboratorio de Micología, Facultad de Ciencias, Universidad Nacional Jorge Basadre Grohman, Miraflores S/N, Tacna 23000, Peru; rubiqm@unjbg.edu.pe; 2 Laboratorio de Biología Molecular de Plantas, Facultad de Ciencias Agronómicas, Universidad de Tarapacá, Gral Velasquez 1775, Arica 1000000, Chile; whuanca@academicos.uta.cl; 3 Facultad de Ciencias, Universidad Nacional Autónoma de México, Ciudad Universitaria, Coyoacán, Ciudad de México 04510, Mexico; 4 Departamento de Recursos Ambientales, Facultad de Ciencias Agronómicas, Universidad de Tarapacá, Avda. General Velásquez 1775, Arica 1000000, Chile; pmunozt@academicos.uta.cl

**Keywords:** oregano, crown and root rot, *Dactylonectria*, *Fusarium*

## Abstract

Oregano (*Origanum vulgare*) cultivation is of great economic importance in Peru. Tacna stands out as its main producer. However, the presence of phytopathogenic fungi represents a challenge for its production. This study aimed to characterize both the morphological and molecular levels of the causal agent of crown and root rot in a crop field in the Camilaca district, Candarave, Tacna. To this end, systematic sampling was carried out using the five-gold method, collecting plants with typical symptoms. Fungi were isolated from diseased roots and characterized using macroscopic and microscopic morphological analysis as well as sequencing and multilocus phylogenetic analysis (ITS, 28S, HIS3, TEF1, TUB2). In addition, pathogenicity tests were performed on healthy plants to confirm the infectivity of the isolates. The results demonstrated that root rot was caused by a complex of phytopathogenic fungi through phylogenetic analysis of *Dactylonectria torresensis*, *Fusarium oxysporum*, *F. iranicum*, and *F. redolens*. These findings represent the first report of these species as causal agents of oregano root rot in Peru, highlighting the need for integrated management strategies that reduce the economic impact of these diseases and contribute to the sustainability of the crop in key producing regions such as Tacna.

## 1. Introduction

Oregano (*Origanum vulgare*), an aromatic plant of the Lamiaceae family, is a crop of great economic importance worldwide, due to its economic, medicinal, and environmental value [1]. Its world production is estimated to reach approximately 15,000 t, with Turkey being the largest producer, followed by Mexico, while in Latin America, Peru leads in production with a growing export, where the Department of Tacna stands out with 2133 t [2,3,4]. In 2023, the harvested area in Tacna reached 2085 ha, with an 8.7% decrease in yield compared to 2022, from 11,147 t to 10,182 t [4].

Various oregano morphotypes are cultivated in this region, including “improved,” “chinito,” “early flowering 1,” “early flowering 2,” “common nigra,” “elephant ear,” “Italian,” and “cocotea,” with “improved nigra,” “chinito,” and “elephant ear” being among the most commercially available [5,6]. These cultivars not only have cultural value but also represent a key source of income for small producers in southern Peru.

The phytosanitary situation of this crop is complex, and one of the factors limiting production is root diseases caused by phytopathogenic fungi. The identification of the fungal complex associated with root problems has not been clear, making it difficult to define effective disease management strategies. Therefore, it is essential to identify the causative agents of the disease.

Although studies on phytopathogens in oregano are limited, research such as that conducted in the Lublin region of Poland has reported the presence of *Fusarium* spp. and *Rhizoctonia solani* in two-year-old plantations, associated with characteristic symptoms such as necrotic leaf spots, progressive wilting, and premature defoliation, significantly affecting crop productivity [7]. Similarly, in Argentina, *F. proliferatum* was reported in oregano plants with symptoms of generalized yellowing, wilting, necrosis of leaves, roots, and crown, as well as defoliation and death of the plant, in the Traslasierra Valley [8]. Likewise, in Peru, a descriptive study in the Cajamarca region reported the presence of *Fusarium oxysporum*, *Fusarium moniliforme*, and *Fusarium roseum* in plants with root rot [9].

Due to the scarcity of updated information on oregano, it was necessary to consider background in agricultural crops where these fungi have been widely studied. *Fusarium* species are polyphagous that affect various crops of agricultural importance [10]. *Fusarium oxysporum* has been associated with root rot of field pea, North Dakota, causing 35% to 75% yield losses, affecting a crop that generated USD 94 million in 2022 [11], and in onion and garlic (*F. oxysporum* f. sp. *cepae*, *F. culmorum*), with reductions of up to 40% in production [12,13]. On the other hand, *Fusarium redolens* caused root rot in American ginseng fields in China with an incidence of 70% in continuous crops and 20% in new fields, severely affecting production [14]. However, there are no reports of *Fusarium iranicum* acting as a phytopathogen in plants. *Dactylonectria torresensis*, on the other hand, is part of the fungal complex responsible for blackleg disease in grapevines and is the most common species in Italy, Portugal, and Spain [15,16,17]. Furthermore, this species is widely distributed in agricultural soils and is recognized for its ability to induce devastating diseases in a wide range of crops [18,19,20,21,22].

Its aggressiveness has been evidenced in *Bletilla striata*, a medicinal species in China, where it caused yield losses of 40–65% and a disease incidence of 45–75% during months of high humidity and temperature [20]. In Ecuador, this pathogen was responsible for significant economic losses in blackberry crops, with a recorded incidence of 13.3% in the provinces of Tungurahua and Bolívar [22]. Likewise, in young grape vineyards in the Czech Republic, *D. torresensis* affected up to 30% of the plants, compromising their root development and crop viability [23]. Although the direct economic impact of these species on oregano has not yet been quantified, their behavior in other crops evidences the high destructive potential of these phytopathogens. This information supports the need to conduct specific phytopathological studies on oregano, especially in producing regions such as Tacna, where the crop represents a key source of income for small farmers.

In addition to their wide distribution and aggressiveness in economically important crops, *Fusarium* spp. and other fungi of the *Nectriaceae* family possess sophisticated infection mechanisms that explain their success as pathogens. This family includes several widely distributed phytopathogenic genera, such as *Fusarium*, *Dactylonectria*, and *Ilyonectria*, responsible for diseases in agricultural and forestry crops around the world [24,25]. The *Fusarium* genus is widely distributed and is a common soil saprophyte associated with insects and decaying organic matter. Its wide distribution is due to its ability to adapt to tropical and temperate habitats [26]. Within this genus, some species such as *Fusarium oxysporum* exhibit a hemibiotrophic lifestyle, characterized by an initial biotrophic phase, in which they colonize host tissues without causing obvious damage, followed by a necrotrophic phase in which they produce toxins and cellulolytic enzymes that cause the destruction of plant tissue [27]. *Fusarium oxysporum* is among the five fungal pathogens with the greatest economic impact on agriculture globally [28].

The pathogenic capacity of *Fusarium* spp. is supported by several mechanisms, including the production of hydrolytic enzymes, effector proteins, and mycotoxins. Carbohydrate-active enzymes (CAZymes) play a key role in the degradation of the plant cell wall, considered the first defensive barrier against infection [29,30]. Secreted effectors interfere with plant immune responses and modify essential cellular processes, facilitating host colonization [31]. In addition, many species of the genus produce mycotoxins such as trichothecenes, fumonisins, and zearalenone, which have phytotoxic effects and pose risks to human and animal health [32]. In particular, *F. oxysporum* produces fusarins, fusaric acid, and chrysogin; the latter confers tolerance to adverse environmental conditions, favoring its survival in the soil [33]. On the other hand, *Dactylonectria torresensis* is soil-borne and is found on four continents. The pathogenicity of this fungus is usually mediated by the production of enzymes that degrade plant cell wall polysaccharides, as well as by toxins such as tentoxin, zearalenone, and HC toxin, which can induce chlorosis, root growth inhibition or shoot deformations, even without visible manifestations of necrosis [34]. However, further studies are still required to confirm the central role of these toxins under natural conditions.

Traditionally, fungal taxonomy is based on the morphology of their reproductive structures. However, this approach has limitations, as it does not allow the differentiation of closely related species due to the high phenotypic plasticity influenced by environmental and genetic factors [34,35,36,37]. In this context, molecular characterization has gained relevance as a key tool for a more precise identification [38]. Internal transcribed spacer (ITS) DNA, although widely used, does not offer sufficient resolution to discriminate closely related species within complexes such as *Fusarium* spp. [39,40]. In contrast, the TEF1-α gene, due to its high polymorphism, has proven to be more effective in phylogenetic studies [41,42]. Furthermore, loci such as HIS3 and TUB2 allow the differentiation of species from related genera such as *Cylindrocarpon*, *Ilyonectria*, and *Dactylonectria*, the latter redefined from multilocus analysis [15,18].

Through morphological, phylogenetic, and pathogenicity analyses, this study aimed to identify the fungal species that cause root rot in oregano (*Origanum vulgare*) plants in Candarave, Peru.

## 2. Materials and Methods

### 2.1. Biological Material

In the Camilaca district, Candarave, Tacna, during May 2023, oregano plants were collected with typical symptoms of the disease: yellowing, curling, and wilting of the leaves.

Five collections were made in five oregano plots, using the five-in-one sampling method [43]. One oregano plant was collected from each sampling point, for a total of 25. The samples were labeled and transported under refrigerated conditions to the laboratory for analysis.

### 2.2. Isolation and Purification of Fungi

Roots showing disease symptoms were washed with running water, and 4 cm segments were cut from the advanced disease zone. These segments were disinfected by immersion in 1% sodium hypochlorite solution for 2 min, followed by three rinses with sterile distilled water, and then dried on sterile absorbent paper. Some segments were placed in a humid chamber at 25 °C to induce sporulation. The resulting spores were then inoculated into the center of the Potato Dextrose Agar (PDA) plate. In addition, small 1-cm pieces of the advanced disease zone were cut from the remaining disinfected segments. These were placed in four equidistant spots on PDA plates and incubated at 25 ± 1 °C until fungal growth was observed. The isolated fungi were purified using the single-spore culture technique [44].

### 2.3. Macroscopic and Microscopic Morphological Characterization

Isolates were macroscopically characterized based on colony morphology on PDA plates after 14 days of incubation at 25 °C [45]. Isolates with characteristics of the *Fusarium* genus were subcultured on carnation leaf agar (CLA) medium for microscopic observation of conidia. Isolates were also subcultured on synthetic nutrient-poor agar (SNA) to induce chlamydospores after 14 days of incubation at 25 °C [46,47]. Isolates with characteristics of the *Dactylonectria* genus were identified by observing macroconidia on PDA and microconidia and chlamydospores on SNA [18,48]. Microscopic examination of all isolates used lactophenol blue as the mounting medium. Thirty measurements of each structure were taken using a Nikon Eclipse Si trinocular microscope (Nikon Corporation, Tokio, Japan) Nikon Eclipse Si trinocular microscope. All isolates were identified to the genus level.

### 2.4. DNA Extraction and Amplification

For DNA extraction, mycelium from pure colonies grown on PDA for 15 days at 25 °C was used. Following the manufacturer’s instructions, the sample was placed in a NucleoSpin^®^ Microbial DNA kit (Macherey-Nagel GmbH & Co KG, Düren, Germany) kit tube to obtain DNA from the isolate. DNA integrity was verified using 1.5% agarose gel electrophoresis. The extracted DNA was then stored at −20 °C for future use. This procedure was repeated for each isolate. From the extracted DNA, the internal transcribed spacer (ITS), the D1-D2 domains of the 28S gene, histone 3 (HIS3), translation elongation factor 1-alpha (TEF1), and β-tubulin fragments (TUB2) were amplified. Amplification was performed using the following primer pairs: ITS4 and ITS5 [49], NL1 and NL4 [50], CYLH3F and CYLH3R [51], EF1-728 and EF2 [52,53], and T1 and CYLTUB1R [51,54] (Table S1), respectively, in a ProFlex thermal cycler (Applied Biosystems, Waltham, MA, USA) (Thermo Fisher Scientific Inc., Foster City, CA, USA). Polymerase chain reaction (PCR) was carried out by mixing 12.5 µL of GoTaq^®^ Master Mix (Promega Corporation, Madison, WI, USA), 1 µL of forward primer (10 pmol/µL), 1 µL of reverse primer (10 pmol/µL), 8.5 µL of nuclease-free water, and 2 µL of DNA sample, obtaining a final reaction volume of 25 µL.

PCR was carried out under the following ITS conditions: an initial denaturation at 95 °C for 2 min, followed by 35 cycles of 95 °C for 30 s, annealing at 53 °C for 30 s, initial extension at 72 °C for 1 min, and a final extension at 72 °C for 5 min. Uniform amplification conditions were used for all genes, varying only in annealing temperature, time, and cycling (Table S1). PCR products were separated by 1.5% agarose gel electrophoresis and stained with 4 µL of SYBR Safe DNA gel stain. They were visualized under UV light using a photodocumentation system (omniDOC, Cleaver Scientific) (Thistle Scientific Ltd., Uddingston, Scotland) to observe amplicon size and purity. Amplicons were purified and sequenced in both directions using Sanger sequencing at the Psomagen laboratory in Rockville, MD, USA.

### 2.5. Phylogenetic Analysis

Sequences were visualized, edited, and assembled using Sequencher 5.0.1 (Gene Codes Corporation, Ann Arbor, MI, USA) to obtain consensus sequences for each locus. The sequences obtained in this study and those retrieved from GenBank were aligned for each locus using the MAFFT v.7 web server (Table S2) [55]. Alignments were manually reviewed and corrected as needed using Mesquite v3.40 [56]. Matrices were generated for the five analyzed fragments. For non-coding regions (ITS and 28S), substitution models were calculated using jModelTest (2.1.6) [57]. For each codon in the coding genes (TEF, TUB2, and HIS3), the optimal models were selected according to the Akaike information criterion (AIC) and W-IQ-TREE [58]. Finally, a concatenated matrix of the markers was obtained using Mesquite v3.40 [56]. Phylogenetic trees were constructed using the concatenated matrix through the CIPRES portal [59], employing two probabilistic methods: maximum likelihood (ML) and Bayesian inference (BI). For the ML analysis, RaxML v8.2 [60] was used with 1000 bootstrap replicates.

For BI analysis, MrBayes (3.2.7) [61] was used, running two independent runs of 20,000,000 generations with four chains. Trees were sampled every 1000 generations and the initial trees were discarded using a burn-in value of 5000 generations. The remaining trees were used to generate a majority rule consensus tree. Convergence of the run was verified using Tracer v1.7.2 [62]. The final tree was inspected using FigTree v1.4.4 [63].

### 2.6. Pathogenicity Test

The pathogenicity of the isolates was assessed by inoculating healthy, four-month-old plants of the *Origanum vulgare* var *Nigra*. Inoculation was performed by immersing the plants in a solution of 1 × 10^6^ conidia/mL for 30 min, with three replicates for each isolate, while the control was inoculated with sterile distilled water. The plants were transplanted into 1 kg polyethylene bags containing a sterile mixture of soil and sand (2:1) as a substrate [64]. Symptoms were assessed daily, and irrigation was adjusted as needed. Finally, reisolation was performed on PDA. Specific evaluation scales were developed based on the symptoms observed in the field (Table 1 and Table 2). For statistical analysis, the nonparametric Kruskal–Wallis test, suitable for ordinal severity data, was used.

## 3. Results

### 3.1. Isolation and Characterization of Fungi Associated with Oregano Root Rot

**Figure 1:**
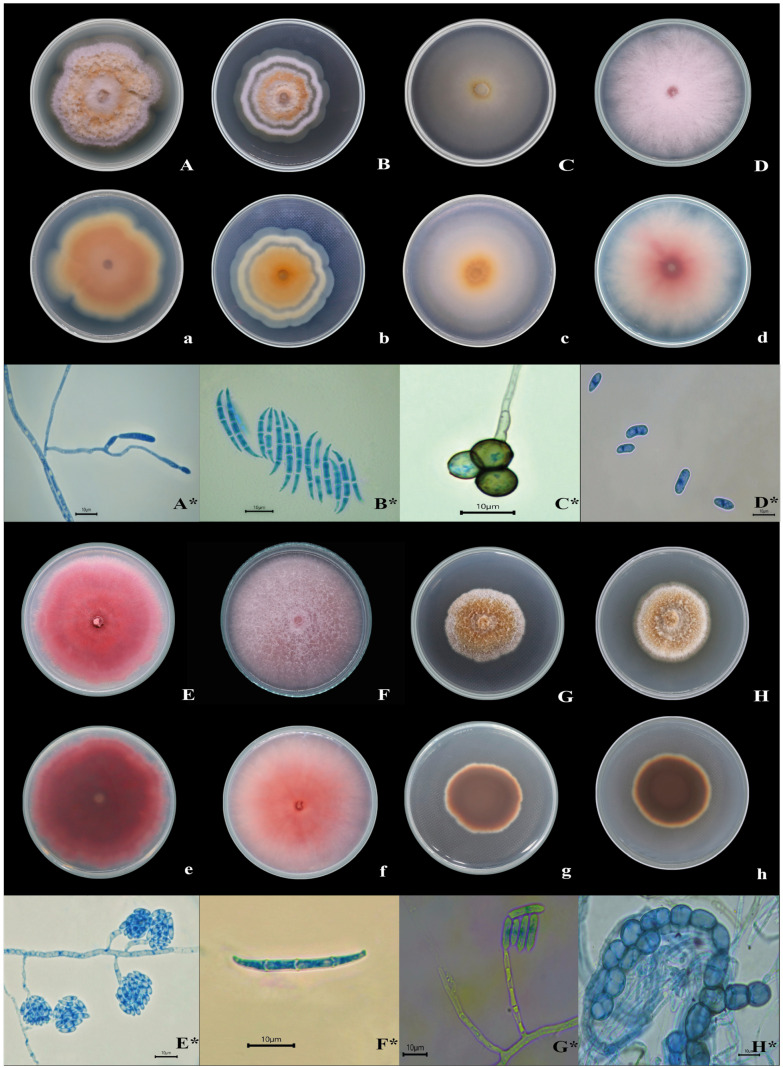
Morphology and cultural characteristics of *Fusarium* spp. and *Dactylonectria* sp. recovered from oregano samples. (Uppercase = upper side of colonies on PDA after 14 days of culture; lowercase = lower side of colonies on PDA after 14 days of culture; Uppercase* = photomicrographs). (**A**,**a**,A*,**B**,**b**,B*,**C**,**c**,C*) = *F. iranicum*, (**D**,**d**,D*) = *F. redolens*, (**E**,**e**,E*,**F**,**f**,F*) = *F. oxysporum*, (**G**,**g**,G*,**H**,**h**,H*) = *D. torresensis*. Scale bar = 10 µm.

A total of 19 isolates were obtained from the five collections of oregano plants with root rot symptoms. Eight morphologically distinct isolates were selected based on macroscopic characteristics, previous research in the scientific literature, and their isolation frequency (Figure 1). These isolates were evaluated for pathogenicity and identified to the species level. The remaining isolates were characterized to the genus level. They are considered common soil saprophytes or potential endophytes and were not inoculated.

The isolates chosen for pathogenicity evaluation, corresponding to the genera *Fusarium* and *Dactylonectria*, were coded and characterized as follows (Table 3). Isolate LUAD03-16 initially exhibited a white colony, which subsequently developed a light pink diffusible pigment with a cottony appearance and opaque texture (Figure 1D,d). The macroconidia measured 22.5–42.2 µm × 3.8–4.4 µm, with a hook-shaped apical cell and a foot-shaped basal cell with three septa. The morphology of the microconidia varied from oval to cylindrical, but were often pointed at one end (Figure 1D*). They were found in simple and branched monophyalides, with a size of 5.1 to 10.2 µm × 2.1 to 3.4 µm. The chlamydospores were terminal or intercalary, spherical or oval, with a smooth wall.

Isolate LUAD03-14 presented a pink, circular colony with an irregular border and a cottony appearance (Figure 1F). In contrast, isolate LUAD03-15 displayed a dark magenta color with a flat, extended, velvety surface (Figure 1E). Microscopically, macroconidia ranged from slightly falcate to straight, with a conical and slightly curved apical cell, while the basal cell was foot-shaped with three septa, measuring between 27.2 and 41.5 × 2.3 and 4.8 µm (Figure 1F*). Microconidia were oval to ellipsoidal, with or without septa, and measured between 5.3 and 10.2 µm × 2.1 and 3.9 µm (Figure 1E*). Chlamydospores were oval and intercalary and measured 4.7–10.3 × 4.8–10.5 µm.

Isolates LUAD03-12, LUAD03-13, and LUAD03-17 exhibited white surface coloration, varying to citrine tones, while maintaining white borders. Isolates LUAD03-12 and LUAD03-13 had a cottony appearance with a well-defined, raised, and lobed border. In contrast, isolate LUAD03-13 displayed concentric rings on its surface (Figure 1B). Isolate LUAD03-17 had a circular shape with an irregular border, a velvety appearance, and a flat, spreading growth pattern (Figure 1C). The macroconidia were dorsoventrally curved, with a slightly elongated apical cell, a rounded to slightly papillate apex, and foot-like basal cells containing 3–4 septa, measuring 27.6–49.7 × 2.5–4.2 µm (Figure 1B*). The chlamydospores were subspherical, with smooth, pale brown walls arranged in clusters or intercalated, and measured 6.9–10.1 × 6.7–10.3 µm (Figure 1C*).

*Dactylonectria* isolates FARU03-10 and FARU03-11 exhibited a colony center with a color ranging from ochre to dark ochre, with abundant aerial hyphae ranging from cream to coral. The colonies had a circular shape, irregular edges and a cottony appearance, and were raised and well-defined (Figure 1G,H). Conidiophores were septate, simple and occasionally branched. Phialids were cylindrical with a narrow top (Figure 1G*). Macroconidia were straight, cylindrical and slightly curved, with a pointed apical cell containing between 1 and 3 septa, measuring 34.2–47.2 × 4.9–5.6 µm. Microconidia were elliptical, aseptate or with a septum, ranging in size from 9.1 to 14.3 × 3.2 to 4.3 µm. Chlamydospores were globose, with slightly thickened and smooth walls, often rough, were found mainly in chains, and measured from 8.1 to 14.5 × 8.3 to 13.6 µm (Figure 1H*).

Morphological characterization of the remaining isolates identified the following genera: *Fusarium* sp1, *Fusarium* sp2, *Trichoderma* sp., *Septonema* sp., *Aspergillus* sp., and *Penicillum* sp. Two unidentified genera and three that did not sporulate were also identified.

### 3.2. Molecular Identification and Phylogenetic Analysis of the Fungi

For isolates FARU0310 and FARU0311, amplicons of approximately 560 bp (ITS), 580 bp (28S), 518 bp (TEFα), 566 bp (TUB2), and 476 bp (HIS3) were obtained. For isolates LUAD0312, LUAD0313, LUAD0316, and LUAD0317, fragments of approximately 505 bp (TEFα), 561 bp (ITS), and 580 bp (28S) were amplified, while for LUAD0314 and LUAD0315, a 501 bp fragment (TEFα) was obtained.

The concatenated matrix used to identify isolates FARU0310 and FARU0311 included the ITS, TEF, 28S, TUB2, and HIS3 loci, covering a total of 3361 base pairs. The default GTRCAT model was applied in maximum likelihood phylogenetic analysis using this concatenated matrix. The following substitution models were also used for Bayesian inference analysis: GTR + I + G for ITS and 28S sequences, K81uf + G for TEF at the first two positions, and GTR + I + G for the third position. For histone, GTR + I + G was used at the first position, while GTR + G was applied to the remaining two positions. Finally, for tubulin, GTR + G, HKY + G, and K81uf + I were applied to the first, second, and third positions, respectively (Table S3).

**Figure 2:**
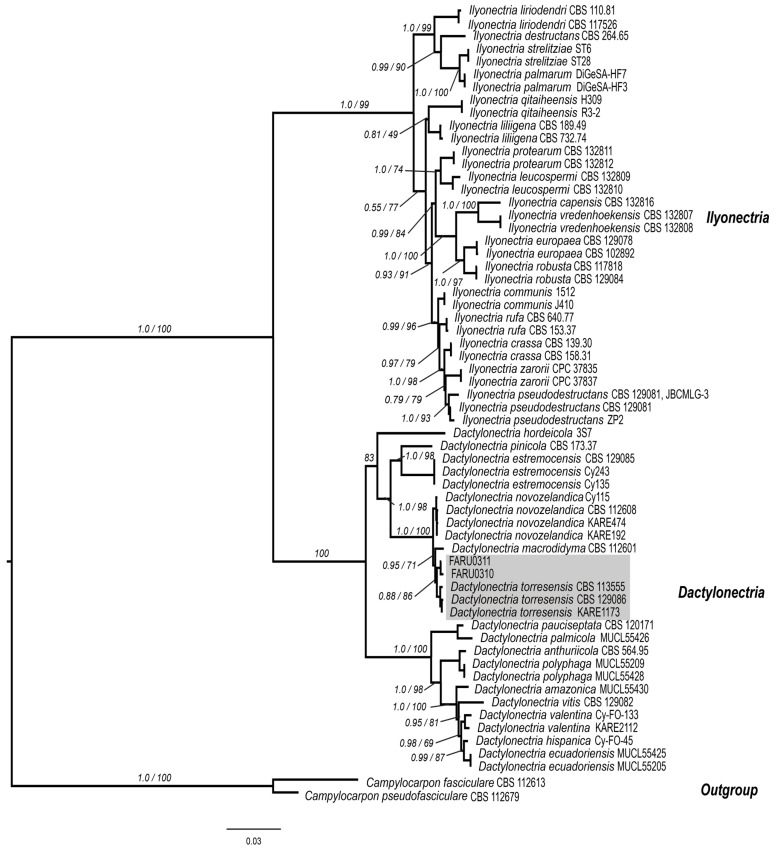
Cladogram of several taxa of the genus *Dactylonectria* with five concatenated markers: ITS, 28S, TEFα, HIS3, and TUB2. The values on the branches represent the Bayesian posterior probabilities and the maximum likelihood bootstrap support values, respectively. Isolates FARU0310 and FARU0311 cluster within the *Dactylonectria* clade, colored in gray, specifically with the species *Dactylonectria torresensis*. The scale bar indicates the number of expected substitutions per site.

Both analysis methods demonstrated that isolates FARU0310 and FARU0311 formed a monophyletic group within the *Dactylonectria torresensis* clade (Figure 2).

**Figure 3:**
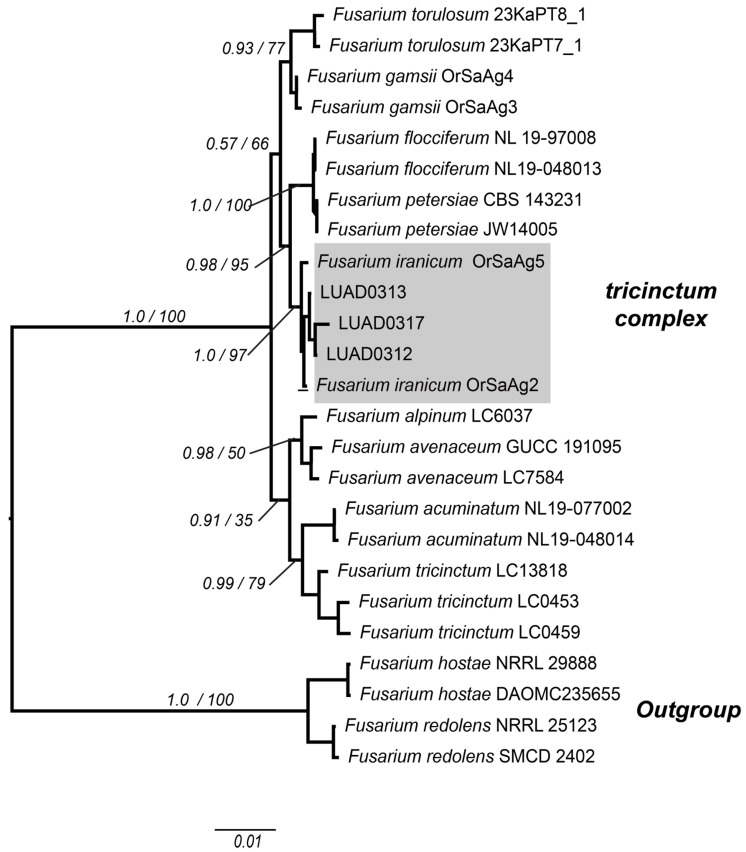
Cladogram of several taxa of the genus *Fusarium* with three concatenated markers: ITS, 28S, and TEF. The values on the branches represent the Bayesian posterior probabilities and maximum likelihood bootstrap support values, respectively. Isolates LUAD0313, LUAD0317, and LUAD0312 cluster within the *Fusarium iranicum* clade, colored in gray. The scale bar indicates the number of expected substitutions per site.

The concatenated matrix of 2064 base pairs, which includes the ITS, 28S, and TEF loci, was used to identify isolates LUAD0312, LUAD0313, and LUAD0317. This matrix was used in the maximum likelihood analysis, where the default GTRCAT model was applied. In the Bayesian inference analysis, the following substitution models were selected: JC + I and HKY for ITS and 28S. In contrast, the GTR + G model was used for the first and third positions of TEF, and the K2P + G model for the second position (Table S3). Within the *Fusarium tricinctum* species complex clade, the three oregano crop isolates LUAD0312, LUAD0313, and LUAD0317 were placed within the *Fusarium iranicum* clade by both probabilistic methods, together with other reference isolates OrSaAg5 and OrSaAg2, with strong support (PP = 1.00; BS = 97%) (Figure 3).

**Figure 4:**
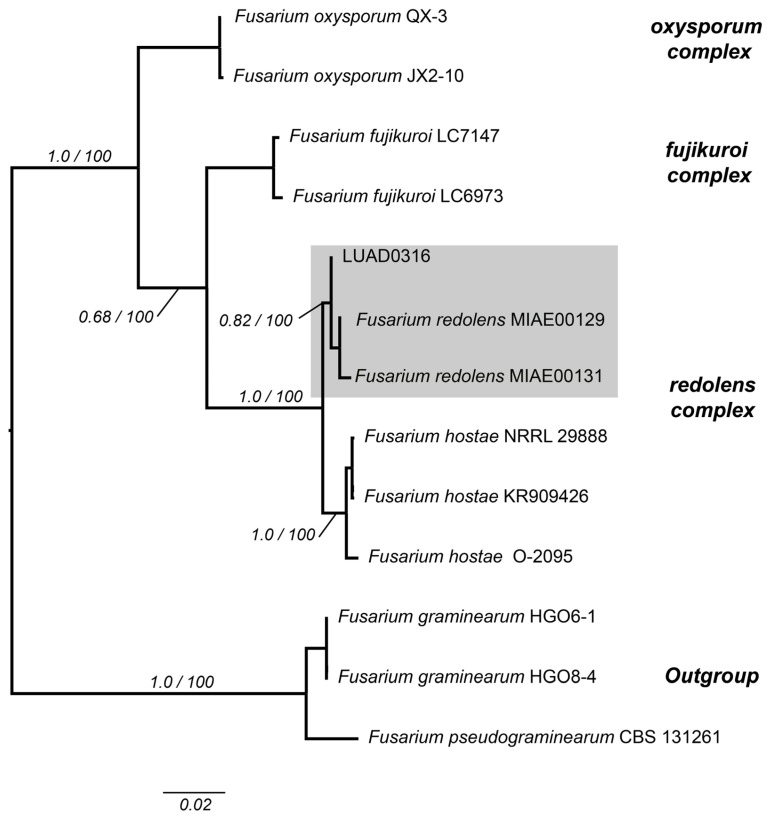
Cladogram of several taxa of the genus *Fusarium* with three concatenated markers: ITS, 28S, and TEF. The values on the branches represent the Bayesian posterior probabilities and maximum likelihood bootstrap support values, respectively. Isolate LUAD0316 clusters within the redolens complex clade, specifically with the species *Fusarium redolens*, colored in gray. The scale bar indicates the number of expected substitutions per site.

The 1856-base-pair concatenated matrix, which includes the ITS, 28S, and TEF loci, was used to identify isolate LUAD0316. The default GTRCAT model was applied in the maximum likelihood phylogenetic analysis using this concatenated matrix. Additionally, the following substitution models were selected for Bayesian analysis: JC + I for ITS and HKY + I for 28S. Specific models for the TEF gene were used: K81uf + I, K81uf + G, and HKY + G for the first, second, and third positions, respectively (Table S3). Isolate LUAD0316 was positioned within the *redolens* complex with strong support (PP = 1.0; BS = 100%) and was associated with the *Fusarium redolens* clade, forming a monophyletic group with strong support (PP = 0.82; BS = 100%) in both probabilistic methods (Figure 4).

**Figure 5:**
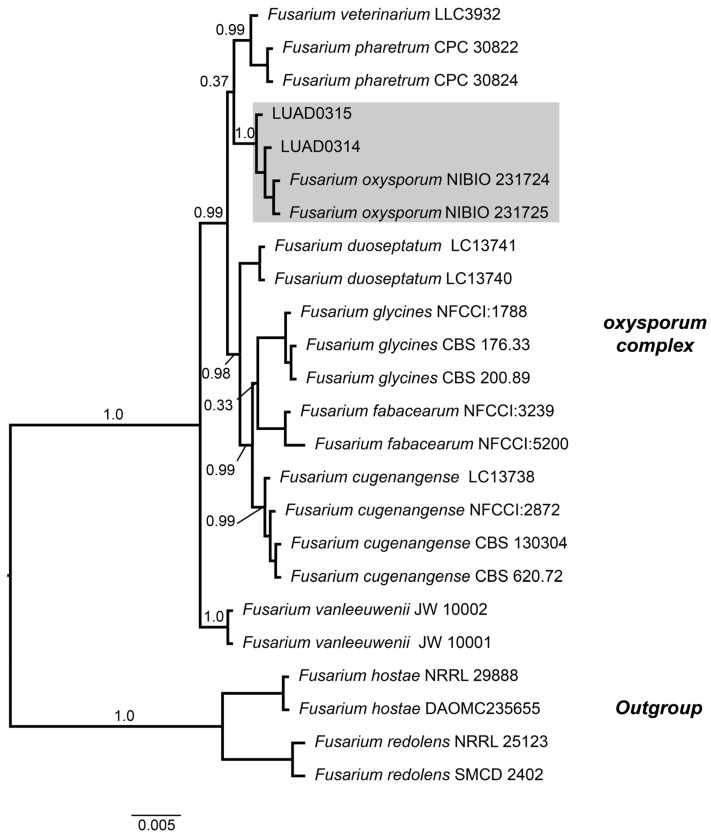
Cladogram of several taxa of the genus *Fusarium* using the TEF locus. Values on the branches represent Bayesian posterior probabilities. Isolates LUAD0314 and LUAD0315 cluster within the *Fusarium oxysporum* clade, colored in gray. The scale bar indicates the number of expected substitutions per site.

Isolates LUAD0314 and LUAD0315 were identified based on a matrix that included the 624-character TEF gene. A Bayesian inference phylogenetic analysis was performed using this matrix with the following substitution models: K2P, HKY, and GTR for the first, second, and third positions of the TEF gene, respectively. Within the *Fusarium oxysporum* complex clade, isolates LUAD0314 and LUAD0315 were positioned within the *Fusarium oxysporum* clade, together with other reference isolates, with strong support (PP = 1.00) (Figure 5).

### 3.3. Pathogenicity Tests

Isolates of *Fusarium oxysporum*, *F. iranicum*, and *Dactylonectria torresensis* caused symptoms in oregano plants such as yellowing, curling, wilting, epinasty, and leaf necrosis, followed by mild defoliation. Notably, only isolates of *F. iranicum* caused death of central branches. *F. redolens*, on the other hand, showed only mild wilting.

**Figure 6:**
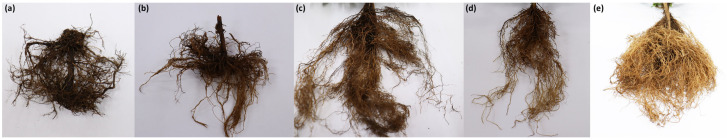
Roots of *Origanum vulgare* plants (Var *Nigra*) inoculated with the isolates and control. (**a**) *Dactylonectria torresensis*, (**b**) *Fusarium oxysporum*, (**c**) *Fusarium iranicum*, (**d**) *Fusarium redolens* and (**e**) control.

**Figure 7:**
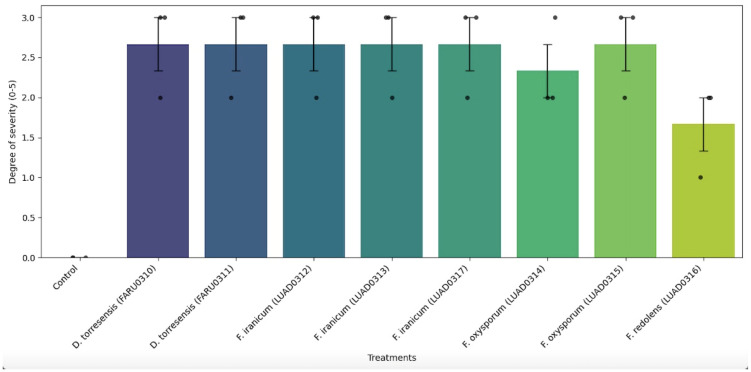
Average severity induced by the eight isolates evaluated in the pathogenicity test.

At the root level, in treatments with *Fusarium oxysporum*, dark brown to black necrosis was observed in the primary root, crown, and some secondary roots. In contrast, isolates of *D. torresensis* exhibited similar symptoms, albeit with black necrosis. In both species, bark sloughing of the secondary root, dark brown necrosis in some rootlets, and vascular discoloration in the crown were recorded. Similarly, plants inoculated with *Fusarium redolens* and *F. iranicum* exhibited brown and dark brown necrosis in the primary root and some secondary roots and rootlets, respectively. The crown showed dark brown necrosis and slight vascular discoloration (Figure 6). Statistical analysis using the Kruskal–Wallis test showed no significant differences in the degrees of severity between treatments (H = 13.92; *p* = 0.0839), indicating that, under the experimental conditions, no quantitative variation was detected between the isolates in terms of their virulence (Figure 7).

## 4. Discussion

This study constitutes the first report of the species *Dactylonectria torresensis*, *Fusarium iranicum*, and *Fusarium redolens* associated with root and crown rot in oregano (*Origanum vulgare*) plants in Peru, specifically in the Tacna region. These findings expand the phytopathological knowledge of the crop by demonstrating that soil-borne genera such as *Dactylonectria* and *Fusarium* play an active pathogenic role in this production area. Although these species have been reported in other hosts, their presence in oregano underscores their adaptive potential and the phytosanitary risk they pose in diverse agricultural systems.

Multilocus phylogenetic analysis allowed for efficient identification of the isolates. ITS sequences confirmed that the isolates belonged to the genus *Fusarium*. However, for more precise discrimination, it was necessary to obtain sequences of the TEF1-α gene, which displays a high degree of sequence polymorphism among closely related species, increasing its effectiveness in inferring deeper evolutionary relationships [65,66].

In the case of *Dactylonectria* spp., the ITS loci 28S, TEF-α, TUB2, and HIS3 were used, allowing for more robust taxonomic delimitation [67]. These results support the use of molecular tools complementary to traditional morphology, especially in fungi with high phenotypic plasticity [35,36,37].

Pathogenicity tests revealed visible differences in severity among isolates. *F. oxysporum* showed moderate severity (grades 2 and 3), while *F. iranicum* and *D. torresensis* reached grade 3, with evident necrosis in the root and crown. However, statistical analysis showed no significant differences (*p* = 0.083). This lack of significance may be due to methodological factors, such as the small size of the replicates and the controlled greenhouse conditions, which possibly attenuated the differential expression of symptoms between treatments. However, it is also possible that the different mechanisms of action of each fungus led to a similar final severity, even though the pathogenic process varies between species.

From a functional perspective, *Fusarium oxysporum* initiates infection by adhering to and penetrating roots, colonizing the bark and progressing to the xylem, where hyphae clog vessels and cause wilting. During infection, it secretes an arsenal of hydrolytic enzymes (CWDEs) such as pectinases, cellulases, and xylanases, which degrade cell walls and facilitate tissue invasion [68]. It also produces toxins such as fusaric acid and trichothecenes, which interfere with host physiology and weaken its defenses. The fungus releases SIX-type effector proteins (Secreted In Xylem), such as SIX1 and SIX4 (Avr1), which suppress host immune responses and determine the specificity of the interaction; these proteins are encoded on accessory chromosomes that allow adaptation to new hosts [68]. Furthermore, *F. oxysporum* produces siderophores such as fusarinin (NRPS6), which are essential for iron uptake and sustaining infection. The virulence of the fungus is regulated by signaling pathways such as MAPK (Fmk1) and cAMP-PKA cascades, as well as G proteins and transcription factors such as FTF1, SGE1, and FOW2, which coordinate the expression of pathogenicity genes. Together, this complex molecular arsenal explains the efficacy of *F. oxysporum* as a vascular pathogen and the similarity in symptom severity among genetically distinct isolates [68].

In the case of *F. redolens*, the severity recorded was similar to that reported in previous studies on American ginseng (*Panax quinquefolium*) [14], ginseng (*P. ginseng*) in China [69], wheat in Kazakhstan [70], and tomato in Algeria [71]. This species has been considered an emerging pathogen, and its detection in oregano suggests a wide host range and adaptability. *F. iranicum*, on the other hand, has not been reported as a phytopathogen; it was described as a species associated with *Agaricus bisporus* and as an endophyte in wheat and grass [47]. This suggests that this fungus could manifest its pathogenicity under specific conditions of stress in the host due to factors such as climate change, water stress, and biological stress [72,73].

The severity of *D. torresensis* is in line with that observed in other crops such as grapevine, strawberry, and baiji, where it has been associated with root rot and decreased plant vigor [19,20,23]. Furthermore, this pathogen is also associated with several fruit trees, olive tree (*Olea europaea* L.), apple (*Malus domestica*), blackberry (*Rubus* sp.), and kiwi (*Actinidia chinensis*) [21,22,74,75]. The observed severity (grade 3) and the induced symptoms reinforce its status as a relevant pathogen. Additionally, some studies have suggested that blackleg fungi have an endophytic phase that can become pathogenic, becoming activated under host stress conditions [76,77,78,79].

The pathogenic capacity and the level of aggressiveness of *Dactylonectria* isolates are mainly linked to the action of enzymes that degrade plant polysaccharides [78]. In addition, it has been reported that *D. torresensis* can produce metabolites such as tentoxin, HC toxin, and zearalenone, with phytotoxic potential [80]. Additionally, some studies suggest that blackleg complex fungi may present an endophytic phase that is activated under host stress conditions, which could explain the variability in the expression of the observed symptoms. In this context, the molecular mechanisms that regulate its virulence are still under study and could play a key role in its phytopathogenic behavior.

From an economic perspective, the Tacna region, the main producing area in Peru, saw a decrease of 965 tons between 2022 and 2023, representing a drop of 8.65% compared to the previous year [81,82]. While this reduction may be due to multiple factors such as climate and agronomic management, the presence of root pathogens confirmed in the field suggests a possible phytosanitary contribution to this loss. Given that these fungi can persist in the soil and affect plantations in successive seasons, their economic impact could increase if preventive and integrated management strategies are not implemented.

As a future guideline, it is recommended to expand the number of replicates. However, the findings presented lay a solid foundation for future research aimed at understanding the epidemiology, integrated management, and risk of spread of these pathogens in oregano-producing areas in southern Peru.

## 5. Conclusions

In this study, through morphological identification, phylogenetic analysis, and pathogenicity tests, *Dactylonectria torresensis*, *Fusarium redolens*, and *Fusarium iranicum* are reported for the first time in Peru as causal agents of crown and root rot in oregano crops, along with the previously reported *Fusarium oxysporum*. These results expand knowledge about the etiology of root diseases in oregano and lay the foundation for the development of integrated management strategies to minimize associated economic losses. Considering that Tacna is the main oregano-producing region in Peru, the accurate identification of these pathogens is key to improving crop health and, thus, ensuring its yield, quality, and export potential. Future research is recommended to investigate the distribution of these fungi in other producing areas, evaluate their interaction with soil and climatic factors, and explore sources of resistance in the different local oregano morphotypes. It will also be important to study the direct economic impact of these diseases in the field and validate effective management strategies, both preventive and curative.

## Figures and Tables

**Table 1:** Severity scale for *Dactylonectria torresensis* and *Fusarium oxysporum*.

Degree	Percentage (%)	Description of the Damage
0	0	Healthy plant.
1	1–20	Less than 5 secondary roots out of 10 evaluated showed dark brown necrosis, and small lesions on the main root, with no impact on the aerial part.
2	21–40	Less than 5 basal leaves with yellowing, curling, and epinasty out of 10 evaluated, with slight defoliation. Necrosis of the main root and more than 8 out of 10 secondary roots with dark brown coloration, necrotic rootlets in brown. Crown with brown necrosis and slight vascular discoloration.
3	41–60	More than 5 basal branches out of 10 evaluated with yellowing, curling of several branches, wilting, epinasty, leaf necrosis, and slight defoliation. Black necrosis in the main root, crown, and more than 5 secondary roots out of 10 evaluated, with bark peeling of the secondary root, dark brown necrosis of rootlets, and vascular discoloration in the crown.
4	61–80	Foliar yellowing of the entire aerial part and defoliation of basal branches. Main root and several secondary roots with black necrosis. Crown with vascular discoloration and black necrosis.
5	81–100	Dead plant.

**Table 2:** Severity scale for *Fusarium iranicum* and *Fusarium redolens*.

Degree	Percentage (%)	Description of the Damage
0	0	Healthy plant.
1	1–20	Small lesions on the main root, with no impact on the aerial part.
2	21–40	Less than 5 secondary roots out of 10 evaluated with brown necrosis and slight discoloration in the crown, with mild wilting.
3	41–60	More than 5 basal leaves out of 10 evaluated with yellowing, curling, mild wilting, epinasty, slight defoliation, and death of central branches. Necrosis of the main root, more than 5 secondary roots, and rootlets out of 10 evaluated with dark brown necrosis. Crown with dark brown necrosis and slight vascular discoloration.
4	61–80	Foliar yellowing of the entire aerial part, defoliation of basal branches, and several dead branches. Main root and several secondary roots with dark brown necrosis. Crown with necrosis and severe vascular discoloration.
5	81–100	Dead plant.

**Table 3:** Fungal isolates obtained from the crown and root of oregano crops in Camilaca, Tacna. The table includes the following columns: Species (taxon identified by morphological and/or molecular characterization), Isolation Code (unique identification assigned to each isolate), and Host (plant organ from which the isolate was obtained).

Species	Isolation Code	Host
*Dactylonectria torresensis*	FARU0310	Oregano Root
*Dactylonectria torresensis*	FARU0311	
*Fusarium iranicum*	LUAD0312	
*Fusarium iranicum*	LUAD0313	
*Fusarium oxysporum*	LUAD0314	Oregano Wreath
*Fusarium oxysporum*	LUAD0315	
*Fusarium redolens*	LUAD0316	
*Fusarium iranicum*	LUAD0317	

## Data Availability

The original contributions presented in this study are included in the article/Supplementary Materials. Further inquiries can be directed to the corresponding authors.

## Supplementary Materials

The following supporting information can be downloaded at: https://www.mdpi.com/article/10.3390/pathogens14080746/s1, Table S1. Primers used in PCR amplification. Table S2. Fungal isolates and GenBank accession numbers used in phylogenetic analysis. Table S3. Selected substitution models for multi-locus phylogenetic analysis.
