# Supplementary material for: Identification of Fungi Causing Root Rot in Oregano Crops in Southern Peru: Morphological and Molecular Analysis
Source: Pathogens. 2025 Jul 29;14(8):746. doi: 10.3390/pathogens14080746 (PMC12389733; doi:10.3390/pathogens14080746)
Supplement: Supplementary file 1 [file pathogens-14-00746-s001.zip › pathogens-3699027-supplementary.pdf]

pathogens

Supplementary tables

Identification of Fungi Causing Root Rot in Oregano Crops in Southern Peru: Morphological and Molecular Analysis

Rubí Adelin Quispe-Mamani [1], Liduvina Sulca-Quispe [1,*], Wilson Huanca-Mamani [2], Mirna G. Garcia-Castillo [3], Patricio Muñoz-Torres [4] and German Sepúlveda-Chavera [4,*]

1 Laboratorio de Micología, Facultad de Ciencias, Universidad Nacional Jorge Basadre Grohman, Miraflores S/N, Tacna 23000, Peru
2 Laboratorio de Biología Molecular de Plantas, Facultad de Ciencias Agronómicas, Universidad de Tarapacá, Gral Velasquez 1775, Arica 1000000, Chile
3 Facultad de Ciencias, Universidad Nacional Autónoma de México, Ciudad Universitaria, Coyoacán, Ciudad de México 04510, Mexico
4 Departamento de Recursos Ambientales, Facultad de Ciencias Agronómicas, Universidad de Tarapacá, Avda. General Velásquez 1775, Arica 1000000, Chile
* Correspondence: lsulcaq@unjbg.edu.pe (L.S.-Q.); gsepulve@uta.cl (G.S.-C.)

Table S1. Primers used in PCR amplification

Gen/Loci	Primers	Sequences	Alignment temperature	Time	Cycles	References
**The elongation factor 1-α (EF1α)**	EF1-728F	CATCGAGAAGTTCGAGAAGG	53 ºC	45 s	40	[52]
	EF2	GGARGTACCAGTSATCATG				[53]
**β-tubulin (Tub2)**	T1	AACATGCGTGAGATTGTAAGT	52 ºC	45 s	40	[54]
	CYLTUB1R	AGTTGTCGGGACGGAAGAG				[51]
**Histone H3 (His3)**	CYLH3F	AGGTCCACTGGTGGCAAG	61ºC	30 s	35	[51]
	C YLH3R	AGCTGGATGTCCTTGGACTG				[51]
**28S**	NL1	GCATATCAATAAGCGGAGGAAAAG	53º C	30 s	35	[50]
	NL4	GGTCCGTGTTTCAAGACG				[50]
**ITS**	ITS4	TCCTCCGCTTATTGATATGC	53 ºC	30 s	35	[49]
	ITS5	GGAAGTAAAAGTCGTAACAAGG				[49]

Table S2. Fungal isolates and GenBank accession numbers used in phylogenetic analysis.

Species	Isolated	GenBank Accesion Nº				
		ITS	*LSU*	*TUB2*	*TEF*	*HIS3*
Campylocarpon fasciculare	CBS 112613	AY677301	HM364313	AY677221	JF735691	JF735502
Campylocarpon pseudofasciculare	CBS 112679	AY677306		KJ022328	JF735692	JF735503
Dactylonectria estremocensis	CBS 129085	MH865182	KM231630	JF735448	JF735806	JF735617
Dactylonectria estremocensis	Cy135				JF735804	JF735615
Dactylonectria estremocensis	Cy243	JF735329		JF735457	JF735815	JF735626
Dactylonectria macrodidyma	CBS 112601	AY677284	KM515899	AY677229	JF735833	JF735644
Dactylonectria novozelandica	CBS 112608, ES 17, ZB3310041	OQ119078	KM515901	OR258684	OR258682	OR258686
Dactylonectria novozelandica	Cy115, WAM158, JZB3310040	OQ119077	MH571719	JF735460	JF735823	JF735634
Dactylonectria novozelandica	KARE192, WAM186, JZB3310039	OQ119076	MH571722	MK409874	MK409951	MK409909
Dactylonectria novozelandica	KARE474, WAM180, JZB3310038	OQ119075	MH571721	MK409875	MK409952	MK409910
Dactylonectria torresensis	CBS 129086	JF735362	KM231631	JF735492	JF735870	JF735681
Dactylonectria torresensis	CBS 113555	JF735350	NG_069231	JF735483	JF735850	JF735661
Dactylonectria torresensis	KARE1173, N37IL	MK400298	KP411808	MK409865	MK409942	MK409900
Dactylonectria torresensis	FARU0310	PV576093	PV576109	PV613359	PV613361	PV613357
Dactylonectria torresensis	FARU0311	PV576094	PV576110	PV613360	PV613362	PV613358
Dactylonectria hordeicola	3S7, EFA 443	MF350482	MF467250	MF350428	MF350509	MF350455
Dactylonectria pauciseptata	CBS 120171	EF607089	KM515903	EF607066	JF735776	JF735587
Dactylonectria pinicola	CBS 173.37	JF735319	KM515905	JF735447	JF735803	JF735614
Dactylonectria vitis	CBS 129082	MH865179	KM515907	JF735431	JF735769	JF735580
Dactylonectria anthuriicola	CBS 564.95	NR_121494	KM515897	JF735430	JF735768	JF735579
Dactylonectria ecuadoriensis	MUCL55425	MF683705	MF683725	MF683642	MF683663	MF683684
Dactylonectria ecuadoriensis	MUCL55205	MF683700	MF683720	MF683637	MF683658	MF683679

Dactylonectria hispanica	Cy-FO-45	KY676882		KY676876	KY676870	KY676864
Dactylonectria valentina	Cy-FO-133	KY676881		KY676875	KY676869	KY676863
Dactylonectria valentina	KARE2112	MK400314		MK409881	MK409958	MK409916
Dactylonectria palmicola	MUCL55426	MF683708	MF683728	MF683645	MF683666	MF683687
Dactylonectria amazonica	MUCL55430	MF683706	MF683726	MF683643	MF683664	MF683685
Dactylonectria polyphaga	MUCL55209	MF683689	MF683710	MF683626	MF683647	MF683668
Dactylonectria polyphaga	MUCL55428	MF683692	MF683713	MF683629	MF683650	MF683671
Ilyonectria capensis	CBS 132816	JX231160	KM515909	JX231112	JX231128	JX231144
Ilyonectria communis	1512	MF350456		MF350402	MF350483	MF350429
Ilyonectria communis	J410	MF350457		MF350403	MF350484	MF350430
Iilyonectria crassa	CBS 139.30, CBS 129083, NW-FVA1829	ON853909	KM515912	JF735393	JF735723	JF735534
Ilyonectria crassa	CBS 158.31	JF735276	KM515911	JF735394	JF735724	JF735535
Ilyonectria destructans	CBS 264.65	AY677273	KM515927	AY677256	JF735695	JF735506
Ilyonectria europaea	CBS 129078, CBS 537.92	MH865175	KM515914	JF735421	JF735756	JF735567
Ilyonectria europaea	CBS 102892	JF735295		JF735422	JF735758	JF735569
Ilyonectria leucospermi	CBS 132809	JX231161	KM515917	JX231113	JX231129	JX231145
Ilyonectria leucospermi	CBS 132810	JX231162	KM515918	JX231114	JX231130	JX231146
Ilyonectria liliigena	CBS 189.49	PP527753	KM515919	JF735425	JF735762	JF735573
Ilyonectria liliigena	CBS 732.74	JF735298	KM515920	JF735426	JF735763	JF735574
Ilyonectria liriodendri	CBS 110.81	MH861308	KM515921	DQ178170	JF735696	JF735507
Ilyonectria liriodendri	CBS 117526	DQ178164		DQ178171	JF735697	JF735508
Ilyonectria palmarum	DiGeSA-HF7	HF937432		HF922609	HF922615	HF922621
Ilyonectria palmarum	DiGeSA-HF3	HF937431		HF922608	HF922614	HF922620
Ilyonectria protearum	CBS 132811	JX231157	MH878255	JX231109	JX231125	JX231141
Ilyonectria protearum	CBS 132812	JX231165		JX231117	JX231133	JX231149
Ilyonectria pseudodestructans	CBS 129081, JBCMLG-3	MH865178	KM515926	MN101796	MN101818	MN105733
Ilyonectria pseudodestructans	ZP2	MT678561		MT810734	MT800962	MT800945
Ilyonectria pseudodestructans	CBS 129081	MH865178	KM515926		JF735752	JF735563

Ilyonectria qitaiheensis	H309	MF350472		MF350418	MF350499	MF350445
Ilyonectria qitaiheensis	R3-2	MT678569		MT810742	MT800970	MT800953
Ilyonectria robusta	CBS 117818	JF735267		JF735382	JF735712	JF735523
Ilyonectria robusta	CBS 129084	JF735273	MH876619	JF735391	JF735721	JF735532
Ilyonectria rufa	CBS 640.77	JF735277	KM515930	JF735399	JF735731	JF735542
Ilyonectria rufa	CBS 153.37	MH855863			JF735729	JF735540
Ilyonectria strelitziae	ST6	KY304649	KY304674	KY304755	KY304727	KY304621
Ilyonectria strelitziae	ST28	KY304671	KY304675		KY304728	KY304643
Ilyonectria vredenhoekensis	CBS 132807	JX231155	NG_070048	JX231107	JX231123	JX231139
Ilyonectria vredenhoekensis	CBS 132808	JX231159	MH878253	JX231111	JX231127	JX231143
Ilyonectria zarorii	CPC 37835	MW114893	NG_079556	MW119263	MW119261	MW119259
Ilyonectria zarorii	CPC 37837	MW114894		MW119264	MW119262	MW119260
Fusarium torulosum	23KaPT8_1, Dl01	PP087417	KC292842		PP094241	
Fusarium torulosum	23KaPT7_1	PP087416			PP094243	
Fusarium acuminatum	NL19-077002	MZ890557	MZ890413		MZ921910	
Fusarium acuminatum	NL19-048014	MZ890556	MZ890412		MZ921909	
Fusarium alpinum	LC6037	MW016687			MW620148	
Fusarium avenaceum	GUCC 191095	MZ724838	OR039362		OR043880	
Fusarium avenaceum	LC7584, SICAUCC 18-0001	MW016674	MK392034		MW620135	
Fusarium flocciferum	NL19-97008	MZ890564	MZ890422		MZ921917	
Fusarium flocciferum	NL19-048013	MZ890563	MZ890421		MZ921916	
Fusarium gamsii	OrSaAg4	LT970824	LT970824		LT970788	
Fusarium gamsii	OrSaAg3	LT970823	LT970823		LT970787	
Fusarium iranicum	OrSaAg2	LT970821	LT970821		LT970785	
Fusarium iranicum	OrSaAg5	LT970822	LT970822		LT970786	
Fusarium iranicum	LUAD0312	PV576095	PV576111		PV613363	
Fusarium iranicum	LUAD0313	PV576096	PV576112		PV613364	
Fusarium iranicum	LUAD0317	PV576097	PV576113		PV613365	

Fusarium tricinctum	LC13818, F749	MW016692	KM249103	MW620153
Fusarium tricinctum	LC0453, F748	MW016690	KM249102	MW620151
Fusarium tricinctum	LC0459, F747	MW016691	KM249101	MW620152
Fusarium petersiae	CBS 143231	MG386078	MG386131	MG386159
Fusarium petersiae	JW14005	MG386079	MG386132	MG386160
Fusarium redolens	NRRL 25123	MW397140		JF740748
Fusarium redolens	SMCD 2402	MW397142		JF272612
Fusarium redolens	LUAD0316	PV576098	PV576114	PV613366
Fusarium hostae	DAOMC235655	KR909426		KR909346
Fusarium hostae	NRRL 29888	NR_171109		MT409455
Fusarium redolens	MIAE00129	HM584896	HQ147594	HM584900
Fusarium redolens	MIAE00131	HM584897	U88113	HM584901
Fusarium hostae	O-2095			AF331822
Fusarium graminearum	HGO6-1	ON416876	AB084297	ON398958
Fusarium graminearum	HGO8-4	ON416875		ON398957
Fusarium pseudograminearum	CBS 131261	MH865930	MH877363	JQ429338
Fusarium fujikuroi	LC7147	MW016452	AB084300	MW580492
Fusarium fujikuroi	LC6973, NRRL 13620	MW016451	NG_060442	MW580491
Fusarium oxysporum	QX-3, KUSF1404	ON398072	MF136406	OQ148161
Fusarium oxysporum	JX2-10, KUSF501	ON351026	MF136403	OQ130017
Fusarium oxysporum	NIBIO 231724			MW316853
Fusarium oxysporum	NIBIO 231725			MW316854
Fusarium oxysporum	LUAD0314			PV613367
Fusarium oxysporum	LUAD0315			PV613368
Fusarium pharetrum	CPC 30822			MH485042
Fusarium pharetrum	CPC 30824			MH485043
Fusarium veterinarium	LLC3932			OP487249

Fusarium cugenangense	NFCCI:2872	ON032413
Fusarium cugenangense	LC13738	MW594323
Fusarium cugenangense	CBS 130304	MH485012
Fusarium cugenangense	CBS 620.72	MH484970
Fusarium glycines	NFCCI:1788	ON032459
Fusarium glycines	CBS 200.89	MH484979
Fusarium glycines	CBS 176.33	MH484959
Fusarium duoseptatum	LC13741	MW594327
Fusarium duoseptatum	LC13740	MW594326
Fusarium fabacearum	NFCCI:3239	ON032385
Fusarium fabacearum	NFCCI:5200	ON032439
Fusarium vanleeuwenii	JW 10002	MZ921891
Fusarium vanleeuwenii	JW 10001	MZ921890

Table S3. Selected substitution models for multi-locus phylogenetic analysis

	ITS	LSU	TEF			HIS3			TUB2		
			1º	2º	3º	1º	2º	3º	1º	2º	3º
FARU0310	GTR+I+G	GTR+I+G	K81uf+G	K81uf+G	GTR+I+G	GTR+I+G	GTR+G	GTR+G	GTR+G	HKY+G	K81uf+I
FARU0311	GTR+I+G	GTR+I+G	K81uf+G	K81uf+G	GTR+I+G	GTR+I+G	GTR+G	GTR+G	GTR+G	HKY+G	K81uf+I
LUAD0312	JC+I	HKY	GTR+G	K2P+G	GTR+G						
LUAD0313	JC+I	HKY	GTR+G	K2P+G	GTR+G						
LUAD0314			K2P	HKY	GTR						
LUAD0315			K2P	HKY	GTR						
LUAD0316	JC + I	HKY + I	K81uf+I	K81uf+G	HKY+G						
LUAD0317	JC+I	HKY	GTR+G	K2P+G	GTR+G						
